# Clinically relevant glioblastoma patient-derived xenograft models to guide drug development and identify molecular signatures

**DOI:** 10.3389/fonc.2023.1129627

**Published:** 2023-04-11

**Authors:** Joshua Alcaniz, Lars Winkler, Mathias Dahlmann, Michael Becker, Andrea Orthmann, Johannes Haybaeck, Stefanie Krassnig, Christina Skofler, Tobias Kratzsch, Susanne A. Kuhn, Andreas Jödicke, Michael Linnebacher, Iduna Fichtner, Wolfgang Walther, Jens Hoffmann

**Affiliations:** ^1^ Experimental Pharmacology and Oncology GmbH, Berlin, Germany; ^2^ Department of Neuropathology, Diagnostic & Research Center for Molecular BioMedicine, Institute of Pathology, Medical University of Graz, Graz, Austria; ^3^ Center for Biomarker Research in Medicine, Graz, Austria; ^4^ Institute of Pathology, Neuropathology, and Molecular Pathology, Medical University of Innsbruck, Innsbruck, Austria; ^5^ Department of Neurosurgery, Charité Universitätsmedizin, Berlin, Germany; ^6^ Department of Neurosurgery, Ernst von Bergmann Hospital, Potsdam, Germany; ^7^ Department of Neurosurgery, Vivantes Hospital Berlin Neukölln, Berlin, Germany; ^8^ Department of Surgery, Molecular Oncology and Immunotherapy, University Medical Center Rostock, Rostock, Germany; ^9^ Max Delbrück Center for Molecular Medicine, Berlin, Germany; ^10^ Experimental and Clinical Research Center, Charité Universitätsmedizin, Berlin, Germany

**Keywords:** glioblastoma, glioma, patient-derived xenograft (PDX), preclinical oncology, drug efficacy, targeted therapy, blood-brain barrier, mTORC1

## Abstract

Glioblastoma (GBM) heterogeneity, aggressiveness and infiltrative growth drastically limit success of current standard of care drugs and efficacy of various new therapeutic approaches. There is a need for new therapies and models reflecting the complex biology of these tumors to analyze the molecular mechanisms of tumor formation and resistance, as well as to identify new therapeutic targets. We established and screened a panel of 26 patient-derived subcutaneous (s.c.) xenograft (PDX) GBM models on immunodeficient mice, of which 15 were also established as orthotopic models. Sensitivity toward a drug panel, selected for their different modes of action, was determined. Best treatment responses were observed for standard of care temozolomide, irinotecan and bevacizumab. Matching orthotopic models frequently show reduced sensitivity, as the blood-brain barrier limits crossing of the drugs to the GBM. Molecular characterization of 23 PDX identified all of them as IDH-wt (R132) with frequent mutations in EGFR, TP53, FAT1, and within the PI3K/Akt/mTOR pathway. Their expression profiles resemble proposed molecular GBM subtypes mesenchymal, proneural and classical, with pronounced clustering for gene sets related to angiogenesis and MAPK signaling. Subsequent gene set enrichment analysis identified hallmark gene sets of hypoxia and mTORC1 signaling as enriched in temozolomide resistant PDX. In models sensitive for mTOR inhibitor everolimus, hypoxia-related gene sets reactive oxygen species pathway and angiogenesis were enriched. Our results highlight how our platform of s.c. GBM PDX can reflect the complex, heterogeneous biology of GBM. Combined with transcriptome analyses, it is a valuable tool in identification of molecular signatures correlating with monitored responses. Available matching orthotopic PDX models can be used to assess the impact of the tumor microenvironment and blood-brain barrier on efficacy. Our GBM PDX panel therefore represents a valuable platform for screening regarding molecular markers and pharmacologically active drugs, as well as optimizing delivery of active drugs to the tumor.

## 1 Introduction

Glioblastoma (GBM) is the most common brain intrinsic neoplasia of the central nervous system, characterized by diffuse and infiltrative growth. About 90% of GBMs are diagnosed *de novo* and test negative for isocitrate dehydrogenase (IDH) 1 mutation R132H. Based on the 2016 WHO classification, they are considered a separate entity of brain tumors, IDH-wildtype (wt) GBM (1, 2). Currently available standard of care is resection, followed by radio-chemotherapy with alkylating agent temozolomide (3, 4). Patients with a high methylation status (> 20%) of the promoter region of O6-methylguanine-DNA methyltransferase (MGMT) show significantly better treatment responses toward temozolomide and better overall survival (5, 6). Another FDA approved drug for recurring GBM is the humanized antibody bevacizumab binding the circulating vascular endothelial growth factor A (VEGF-A). However, despite its successful use for therapy of other cancers (7), it did not reach the same efficacy in GBM. Combination of bevacizumab with temozolomide in recurring GBM did improve patients’ progression free survival and quality of life, but not overall survival (8, 9).

Molecularly, IDH-wt GBM are characterized by frequent mutations in TP53 and phosphatase and tension homologue (PTEN), as well as by epidermal growth factor receptor (EGFR) mutation and amplification (10). Deregulations in PTEN/PI3K/Akt/mTOR signaling, including increased receptor tyrosine kinase signaling, occur in about 80% of GBM (11). Analysis of transcriptional features furthermore revealed different molecular GBM subtypes, hinting towards different tumor evolution and biology within the group of IDH-wt GBM (12, 13). Still, their potential use as predictive markers or targets remains limited. The high heterogeneity of GBM and redundant activation of downstream signaling pathways suggest targeting one molecule alone might be insufficient to improve patients’ outcome (14–17).

To better understand GBM biology and resistance formation, PDX models have been a valuable tool in preclinical and translational research. Subcutaneous (s.c.) GBM PDX have been shown to closely resemble the original tumor’s morphology and molecular heterogeneity, and they thus allow for the testing of new treatment approaches on a patient individual basis (18–21). However, since these models are not able to recapitulate critical physiological structures like the blood-brain barrier (BBB) and its potential to limit availability of anti-cancer compounds within the brain, orthotopic PDX represent a crucial addition in assessment of treatment efficacy and optimization (22).

In this study we aimed to establish a well characterized panel of GBM PDX of matching s.c. and orthotopic models. Analysis of chemosensitivity, as well as the available molecular data on gene expression and mutation allows for identification of molecular signatures and markers, and for identification of advantageous treatment regimens and combinations. Orthotopic PDX allow validation in a model more closely resembling the GBM physiological tumor microenvironment within the central nervous system and the BBB’s impact on drug efficacy (23).

## 2 Materials and methods

### 2.1 Patient tumors

Tumor tissue was collected directly during surgery. Patients included in the study gave written informed consent and specimen collection was approved by the local Institutional Review Board of Charité University Medicine, Germany (EA4/019/12), the Ethics Committee of the University of Rostock, Germany (A 2009/34) and the Institutional Ethics Committee of the Medical University of Graz, Austria (24-402 ex 11/12).

### 2.2 Generation of s.c. PDX GBM models

Animal studies were performed in accordance with the German Animal Welfare Act and approved by local authorities (Landesamt für Gesundheit und Soziales, LaGeSo Berlin, Germany) under the permission H0308/18 for the PDX generation and proliferation *in vivo* and A0010/19 for *in vivo* therapy experiments. For establishment of s.c. PDX models, patient tumor specimens were cut into 3-4 mm sized fragments and transplanted s.c. on anesthetized NOD.Cg-Prkdcscid Il2rgtm1Wjl/SzJ (NSG) mice (Charles River Laboratories, Sulzfeld, Germany). Health status of mice was checked daily, and body weight and tumor size were measured twice per week. When tumors successfully engrafted and reached a size of about 1 cm³, they were excised, cut into 3×3×3 mm sized fragments and transplanted s.c. on female NMRI Foxn1nu mice for the consecutive *in vivo* passage. Mice were housed under standard conditions in IVC caging systems with 22°C +/- 1°C, 12 h light-dark cycle, food, water and nesting material *ad libitum*.

### 2.3 Generation of orthotopic PDX GBM models

PDX tumor tissue obtained from s.c. *in vivo* passage (passage numbers between 4 and 9) was used to prepare a single cell suspension *via* mechanical break up (gentleMACS Dissociator, Miltenyi Biotec, Teterow, Germany). Anesthetized mice were fixed in a stereotactic frame, the skin on the scull was opened and 2 µl cell suspension of 1×10^5^ tumor cells was injected intracerebral (i.cer.) into the cortex of the right hemisphere. After cell injection, the syringe was removed slowly, and the skin was closed using Histoacryl ^®^ tissue adhesive (B.Braun, Melsungen, Germany). The following day mice received meloxicam subcutaneously.

Animal health condition was checked daily and body weight was measured at least twice weekly. Mice were terminated for ethical reasons when showing behavioral abnormalities and body weight loss > 10%; both signs for progressive intracranial tumor growth. Brains were dissected and frozen in isopentane at -80°C. Sequential 10 µm cryostat microtome sections in coronal plane were prepared, cresyl violet-stained and the tumor area measured as described previously (18, 24).

### 2.4 Chemosensitivity testing of GBM PDX

For sensitivity testing of s.c. PDX models, 3×3×3 mm tumor fragments (passage numbers between 2 and 6) were transplanted subcutaneously onto female Rj : NMRI-Foxn1nu/nu nude mice (Janvier Labs, Le Genest-Saint-Isle, France) as described (see above). Animals were randomized and treated at palpable tumor size (0.08 to 0.2 cm³) with the respective drugs: bevacizumab (10 mg/kg intraperitoneally, 3× per week for 2 weeks; Roche, Basel, Switzerland), everolimus (5 mg/kg orally, (day 1-5)×2; Novartis, Basel, Switzerland), irinotecan (15 mg/kg intraperitoneally, (day 1-5)×2; Fresenius, Bad Homburg, Germany), salinomycin (10 mg/kg orally, day 1-14; Sigma-Aldrich, St. Louis, MI, USA), Sorafenib (80 mg/kg, orally, (day 1-5)×2; Bayer, Leverkusen, Germany) and temozolomide (90 mg/kg orally, day 1-5; MSD, Kenilworth, NJ, USA). Drug efficacy was determined by measurement of tumor volumes (TV). TV measurement was performed with a digital caliper and volumes were calculated using the formula:


$$TV\;\left[cm^{3}\right]=\frac{length\;\times \;width^{2}}{2}$$

Studies were terminated for ethical reasons when first animals reached a TV > 1.5 cm³. To describe therapeutic responses, the ratio (T/C, in %) of mean volumes of tumors in treatment (T) versus control groups (C), as well as relative tumor volumes (RTV) in treatment groups were used:


$$RTVx\;\left[\%\right]=\frac{TVx}{TVo}\times 100$$

A T/C value > 50% was defined as no response, > 25% as minor response, > 10% as moderate response and T/C ≤ 10% a strong response. We furthermore considered a RTV value > 1.7 as progression, a RTV > 0.3% stable disease, and RTV ≤ 0.3% as partial remission.

For sensitivity testing of orthotopic GBM PDX models, tumor cells were inoculated, and animals randomly distributed into control and treatment groups. Treatment was started 6 to 7 days after tumor cell inoculation. Once first animals showed health impairments, the study was terminated and maximum tumor area in coronal plane measured microscopically and used to evaluate therapeutic responses.

### 2.5 Statistical analyses

For statistical analyses of tumor size differences between control and treatment groups at study end, one-way ANOVA followed by Dunnett’s multiple comparison test were performed. Prism software v5.02 (GraphPad Software, San Diego, USA) was used for all analyses.

### 2.6 Histology and immunohistochemistry of PDX models

For histopathological analysis, 5 µm sections of formalin fixed paraffin embedded (FFPE) tumor samples were deparaffinized in ROTICLEAR^®^ (Carl Roth GmbH, Karlsruhe, Germany) and rehydrated using ethanol and distilled water. Sections were then stained according to a standard hematoxylin-eosin protocol ​ (24)​. For immunohistochemistry (IHC) antigen retrieval was done in hot citrate buffer for 15 min, followed by cooling for 40 min and washing with PBS.

For MGMT staining, slides were blocked with 5% goat serum in PBS for 1 h at room temperature (RT), and subsequently incubated with primary antibody (5 µg/ml rabbit anti-MGMT, PA5-79668, ThermoFisher Scientific, Germany), for 1 h at RT. Sections were then washed twice in TBST buffer (20 mM Tris/HCl pH 7.5, 150 mM NaCl, 0.1% Tween-20) and incubated with the SuperVison 2 Single Species HRP-polymer rabbit kit (DCS GmbH, Hamburg, Germany) according to the manufacturer’s instructions. Sections were counterstained with hematoxylin solution.

For Ki-67 staining, slides were first blocked with 3% hydrogenperoxide for 30 min, washed twice with PBS and then with 5% normal goat serum in PBS for 30 min, both at RT. After blocking, sections were incubated with primary antibody (6.5 µg/ml rabbit anti-Ki-67 in PBS, ab15580, Abcam, Cambridge, UK) for 1 h at RT. Sections were then washed twice in TBST buffer and incubated with HRP-conjugated secondary antibody (#111035003, 1:200, Jackson ImmunoResearch Laboratory, West Grove, USA) for 30 min, RT. Sections were washed twice in TBST, followed by detection of secondary antibody using chromogen substrate buffer DAB+ (#K3468, Dako North America, Inc., Carpinteria, CA, USA) and DAB-chromogen (#K3468, Dako North America, Inc., Carpinteria, CA, USA). Sections were washed with PBS, then with distilled water and counterstained with hematoxylin solution.

All Images were acquired using the Axioskop 40 and AxioVision V3.5 (Zeiss, Jena, Germany).

### 2.7 Molecular characterization of s.c. PDX models by RNA sequencing

RNA sequencing (RNASeq) of untreated s.c. tumor tissue samples was performed for 23 established GBM PDX models. Next-generation sequencing and processing of the raw data was performed by ATLAS Biolabs GmbH (Berlin, Germany).

About 50-100 mg snap frozen PDX tumor tissue was dissolved in 1.5 ml of TRIzol™ (ThermoFisher Scientific, Carlsbad, CA, USA) using a gentle MACS dissociator and M tubes (Miltenyi Biotec, Bergisch Gladbach, Germany). The integrity of the isolated total RNA was analyzed using an Agilent Bioanalyzer 2100 and the RNA 6000 Nano Kit (Agilent, Santa Clara, CA, USA). The Illumina TrueSeq Stranded mRNA Library Prep Kit was used for preparation of RNAseq libraries, followed by 100 bp PE-sequencing on an Illumina HiSeq 2500 device with a depth of 80–100 million reads (40–50 Mio cluster) (Illumina, Cambridge, UK).

### 2.8 Bioinformatic data processing

Read quality was validated with FastQC v0.11.8 (25). Human (reference: ensembl hg38) and mouse (reference: ensembl mm10) reads were split with Xenome v1.0.1 (26). STAR aligner v2.6.1a (27), QualiMap v2.2.1 (28) and eXpress v1.5.1 (29) were used to map the human-specific reads and quantify transcript expression.

GATK 4.0.2.1 (https://github.com/broadinstitute/gatk/releases) (30) and the Ensemble Variant Effect Predictor (VEP), release 94 (https://www.ensembl.org/info/docs/tools/vep/index.html) (31) were used for variant calling and annotation of mapped reads. Additionally, to verify annotations and to further interpret variants of interest the openCRAVAT pipeline v2.2.7 was used (32). The mutational analysis was performed for a set of GBM-relevant genes (33). Quality-tested variant calls were filtered based on allele frequencies from gnomAD 3.1. to identify somatic alterations. Variants not included in gnomAD 3.1 or with a gnomAD allele frequency below 0.001 were considered (**Supplementary Table S1**).

Copy Number Variations (CNV) were predicted from gene expression mutational data using RNAseqCNV (34) and CaSpER (35).

RNAseq raw count data were transformed to gene length corrected trimmed mean of M-values (GeTMM) (36) to perform single-sample gene set enrichment analyses (36) (https://github.com/broadinstitute/ssGSEA2.0) regarding gene sets of the molecular signature database (MsigDB v2022.1) (37). Subtype classification was performed based on published gene set expression profiles (12). Group-wise comparison of enriched gene sets between responding and resistant PDX models towards individual therapy was performed with gene set enrichment analyses v4.3.2 (37).

## 3 Results

### 3.1 Engraftment of PDX models

A total of 131 tumor tissues from patients diagnosed with GBM were used for PDX generation. Of these, 39 models (30%) engrafted and were consecutively passaged subcutaneously for a minimum of three times and were then considered stable, established s.c. PDX. Median age of patients at resection is 63, and 15 of the characterized GBM PDX were from male patients (58%) (**Table 1**), reflecting the clinical situation of GBM grade IV. Until now, 26 GBM PDX models have been subject to chemosensitivity testing, 23 of which subject to molecular characterization by RNA sequencing. These 26 models include 19 GBM, 4 recurrences of GBM and 3 GBMs with unknown treatment history. In addition, 15 GBM PDX were established as orthotopic, intracranial models, of which 4 have been subject to sensitivity testing.

**Table 1:** Clinical characteristics of patients that provided tumor tissue of subcutaneously established and chemosensitivity-tested PDX models.

PDX	Patient		Tumor characteristics	
	sex	age	Diagnosis	Past treatments
Glio10193	male	n.a.	Glioblastoma Grade IV	no
Glio10315	male	n.a.	Glioblastoma Grade IV	no
Glio10485	female	n.a.	Glioblastoma	n.a.
Glio10535	male	n.a.	Glioblastoma Grade IV	no
Glio10618	female	66	Glioblastoma Grade IV	no
Glio10888	male	n.a.	Glioblastoma Grade IV	no
Glio10995	female	n.a.	Glioblastoma	n.a.
Glio11305	male	71	Glioblastoma Grade IV	no
Glio11368	male	49	Glioblastoma Grade IV	no
Glio11413	female	n.a.	Glioblastoma	n.a.
Glio11414	female	60	Glioblastoma Grade IV	recurrence, yes
Glio11415	male	55	Glioblastoma Grade IV	recurrence, n.a.
Glio11575	male	66	Glioblastoma Grade IV	recurrence, n.a.
Glio11874	male	63	Glioblastoma Grade IV	no
Glio12032	male	69	Glioblastoma Grade IV	no
Glio12421	male	61	Gliosarcoma Grade IV	no
Glio12464	female	62	Glioblastoma Grade IV	no
Glio12826	male	53	Glioblastoma Grade IV	no
Glio12827	male	60	Glioblastoma Grade IV	recurrence, n.a.
Glio12856	female	77	Glioblastoma Grade IV	no
Glio13066	male	68	Glioblastoma Grade IV	no
Glio14227B	male	61	Glioblastoma Grade IV	no
Glio15194	female	67	Glioblastoma Grade IV	no
Glio15380B	female	49	Glioblastoma Grade IV	no
Glio15782	female	79	Glioblastoma	no
Glio15807	male	42	Glioblastoma Grade IV	no

n.a., data not available.

### 3.2 Morphology and growth characteristics of PDX over several *in vivo* passages

**Figure 1:**
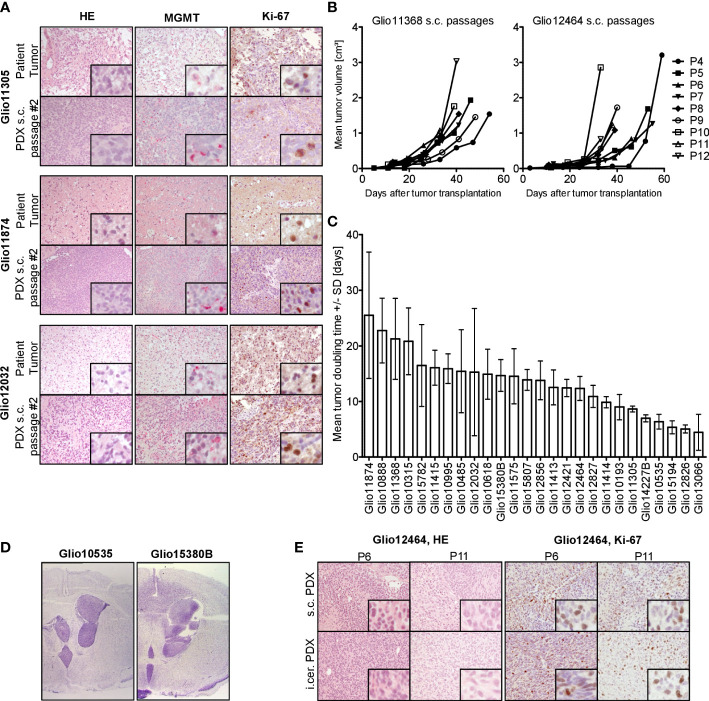
Histological and biological characteristics of GBM PDX. **(A)** Analysis of three representative patient tissue samples and respective s.c. PDX tissue from *in vivo* passage #2 revealed comparable histology (HE), MGMT expression (red staining) and expression of proliferation marker Ki-67 (brown staining), 200-fold magnification, inset 800-fold magnification. **(B)** Comparable growth over several consecutive s.c. passages of PDX models Glio11368 and Glio12464. **(C)** Heterogeneous tumor doubling times in our panel of established PDX models, n=3-6. Mean and standard deviation (SD). **(D)** Comparison of nodular and infiltrative growth in two different orthotopic (intracerebral, i.cer.) PDX models (cresyl violet staining, tumor tissue stained purple) (0.9-fold magnification). **(E)** Analysis of PDX Glio12464 revealed comparable histology and Ki-67 expression over several consecutive s.c. passages and parallel orthotopic inoculation. Examination of Ki-67 expression (proliferation marker) in PDX tumor tissue *via* IHC. Positive areas in the sections are stained brown. 20-fold magnification, inset 160-fold magnification.

Our established GBM PDX models closely resemble the respective patient’s tumor histology, with similar morphology and expression patterns of MGMT and Ki-67 (**Figure 1A**). Expression of proliferation marker Ki-67 was comparable over several s.c. passages and after parallel orthotopic inoculation, as seen in immunohistological stainings of PDX tumor sample sections (**Figure 1E**). Individual orthotopic PDX models were able to retain the characteristic infiltrative growth of GBM (**Figure 1D**), compared to nodular growing s.c. tumors. Furthermore, each model displayed stable characteristic growth patterns over several s.c passages, with a slight increase in tumor growth rate in higher passage numbers (**Figure 1B**). Tumor doubling times varied highly between different s.c. models (**Figure 1C**), reflecting the heterogeneity of our GBM PDX panel.

### 3.3 Chemosensitivity of s.c. GBM PDX models

**Figure 2:**
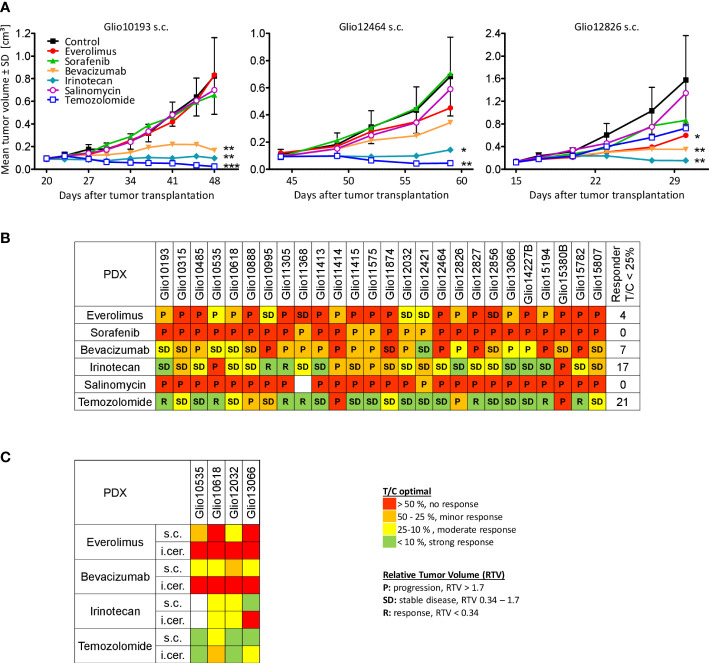
Chemosensitivity of established glioma PDX. **(A)** Examples of drug testings of three s.c. glioma PDX models, illustrating model-specific growth characteristics and treatment responses to different drugs, like temozolomide. N=3-5. Mean and standard deviation (SD). One-way ANOVA, Dunnett’s multiple comparison test. Significant differences to control (PBS) at study end: * p < 0.05, ** p < 0.01, *** p < 0.001. **(B)** Treatment response evaluation as mean tumor volume of treated tumors divided by mean tumor volume of tumors in the corresponding control group (T/C optimal in %) revealed PDX individual sensitivity profiles. In addition, RTV as response criteria is indicated as progression, stable disease or regression in respective groups. N=2-6 per group. **(C)** T/C values at study end of orthotopic PDX models Glio10535, Glio10618, Glio12032 and Glio13066 revealed reduced sensitivities when compared to matching s.c. models. The maximum tumor area in coronal plane was used as measure for i.cer. tumor growth. RTV could not be calculated for orthotopic PDX, as tumor sizes were only measured once at study end.

PDX tumor bearing mice received monotherapies of everolimus, sorafenib, bevacizumab, irinotecan, salinomycin or standard of care temozolomide for up to 2 weeks. Tumor growth was monitored during therapies until the study was terminated for ethical reasons (**Figure 2A**). Of the administered compounds, salinomycin and sorafenib showed no efficacy in our panel of GBM PDX. The anti-VEGF antibody bevacizumab and mTORC1 inhibitor everolimus showed only limited efficacy, leading to moderate (T/C < 25%) to strong responses (T/C < 10%) and stable tumor volumes (RTV 0.34-1.7) in 7 and 4 out of 26 models, respectively. A better response rate was seen for topoisomerase I inhibitor irinotecan, which led to stable tumor volumes in 20 and tumor regression (RTV > 0.34) in 2 models. When compared compared to untreated PDX tumors we only observed a moderate to strong response in 17 models. The standard of care drug temozolomide showed the best tumor growth inhibition among the tested compounds. We observed moderate to strong responses in 21 models, with treatment leading to stable tumor volumes in 15 models and tumor regression in 7 models (**Figure 2B**). Interestingly, Glio11414, a PDX established from a recurrent GBM previously treated with temozolomide, showed resistance toward temozolomide.

### 3.4 Chemosensitivity of orthotopic GBM PDX models

The BBB and the tumor microenvironment within the brain parenchyma significantly contribute to tumor biology and eventually treatment outcome in GBM. We therefore compared efficacy of bevacizumab, everolimus, irinotecan and temozolomide in 4 orthotopic GBM PDX (**Figure 2C**). To evaluate treatment responses between s.c. and orthotopic PDX we used T/C at study end [%].

The targeted drugs bevacizumab and everolimus performed less efficiently in orthotopic PDX tumors compared with matching subcutaneously growing PDX tumors (**Figure S1**). None of the orthotopic models were considered responders to everolimus (T/C > 50%), while it caused a reduction of tumor volumes (T/C < 50%) in two out of four matching s.c. models. For bevacizumab, differences in efficacy in s.c. versus orthotopic models were even more pronounced. All s.c. growing PDX models were considered minor to moderate responders (T/C 50 - 10%), but no response was seen in the four matching orthotopic GBM models. Regarding irinotecan, chemosensitivity of s.c. vs. orthotopic models was different. Irinotecan showed similar efficacy in orthotopic and matching s.c. Glio10618 and Glio12032 models. Compared to their respective vehicle treated control group, orthotopic tumors were significantly reduced in size (**Figures S1B, C**) and showed moderate responses (T/C 10% - 25%). However, in the orthotopic PDX Glio13066, irinotecan showed no efficacy (T/C 84%), whereas it caused pronounced tumor growth inhibition in the matching s.c. model (T/C 7%). Out of the four tested drugs, temozolomide showed best efficacy in the orthotopic PDX, where in all 4 tested models glioma growth was reduced (T/C 2% - 41%), with significant differences to control groups in 2 out of 4 tested models (**Figures S1A–D**). Temozolomide efficacy in s.c. models was slightly better in all models showing moderate to strong responses (T/C 1% - 13%). In conclusion, our diverging results point to the importance of orthotopic GBM PDX models to evaluate drug efficacy in a clinically relevant setting.

### 3.5 Mutation profile of s.c. GBM PDX models

**Figure 3:**
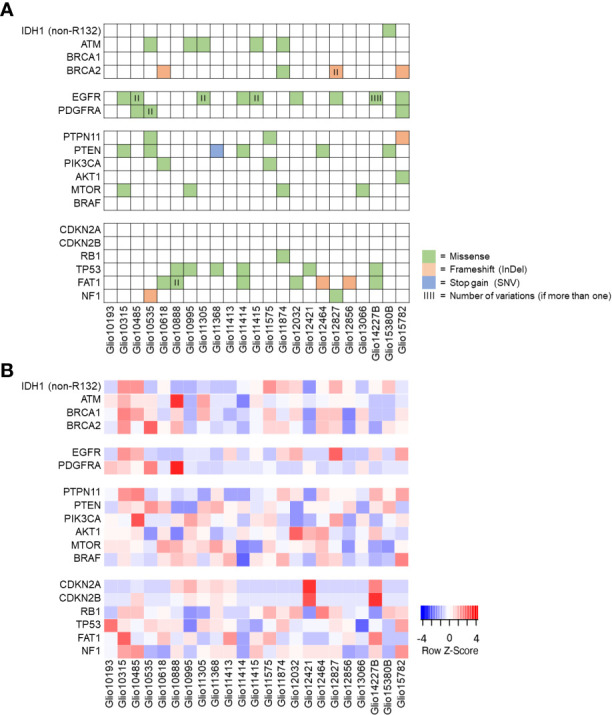
Mutation status of selected genes and their expression in GBM PDX models. **(A)** Using available RNA sequencing data, a selection of genes frequently mutated in GBM was analyzed for mutations in our panel of GBM PDX, revealing PDX individual profiles. **(B)** Expression of the genes listed, with comparably high EGFR expression in most PDX bearing EGFR mutations.

The mutational status of 23 GBM PDX was analyzed using the available RNA sequencing data regarding genes frequently mutated in GBM (**Table S2**). In 23 of these genes, we could identify somatic mutations, with the most frequent mutations occurring in EGFR and PARP1 in 9 PDX models each. In addition, mutations in TP53 (7 PDX), FAT1 and PTEN (6 PDX each), as well as ATM, BRCA2 and MTOR (each in 5 PDX) were identified. Overall, in 12 PDX mutations within the PI3K/Akt/mTOR signaling pathway could be found (**Figure 3A**). We furthermore used available RNA sequencing data to visualize the expression of mutated genes, allowing for selection of PDX expressing specific targets for preclinical studies. Interestingly, 6 of the 9 PDX bearing mutations in EGFR showed a comparably high expression of the mutated receptor (**Figure 3B**).

### 3.6 Molecular GBM PDX subtypes

**Figure 4:**
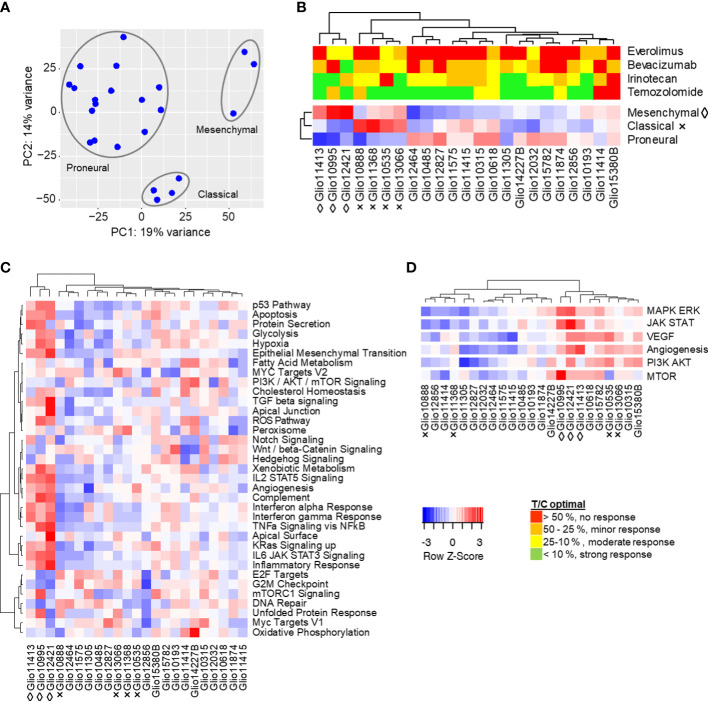
Molecular characteristics of s.c. glioma PDX models. **(A)** Global gene expression of 23 glioma PDX models by principal component analysis. Similarity of transcriptomes is represented by their spacial distribution in the plot, with three custers visible. **(B)** The observed clustering into three groups could be replicated in subsequent analyses of expressions of gene sets characteristic for proposed molecular subtypes mesenchymal (**◊**), classical (**×**) and proneural (no indication). **(C)** Single sample gene set enrichment analysis of glioma PDX models regarding 34 selected hallmarks and clustering of models resembling the mesenchymal subtype. **(D)** Combined enrichment scores of gene sets related to MAPK/Erk, JAK/STAT, VEGF, PI3K/Akt and mTOR signaling, as well as angiogenesis. Gene sets analyzed individually in **Figure S3**. Red (positive Z-score): higher expression of gene set than in the average of all models. Blue (negative Z-score): lower expression.

We performed copy number variation analysis and principal component analysis to determine transcriptomic similarities within the cohort and to identify potential molecular subgroups. While clustering in copy number variation remained inconclusive (**Figure S2**), the principal component analysis revealed two clusters of three and four models, and a third cluster of 16 models. Cluster I consists of Glio10995, Glio12421 and Glio11413, whereas cluster II contains Glio10535, Glio10888, Glio11368 and Glio13066 (**Figure 4A**). To clarify whether the observed clustering is in line with molecular GBM subtypes mesenchymal, classical and proneural, we analyzed a set of 150 genes described as characteristically expressed in these subtypes (**Figure 4B**) (12). Members of cluster I are enriched for mesenchymal signature genes, while members of cluster II are enriched for signature genes of the classical phenotype. The remaining 16 models formed a third cluster defined by comparably high enrichment of genes related to the proposed proneural subtype, but with enrichment in more than one molecular subtype in individual models.

### 3.7 Transcriptome and correlation analysis of s.c. GBM PDX models

To gain further insight into the tumor biology and cancer-related pathways of tumor progression in our PDX models, we compared their transcriptomes according to gene sets of cancer hallmarks (**Figure 4C**). Models of cluster I, the proposed mesenchymal subtype, also clustered in gene sets like epithelial mesenchymal transition, hypoxia, glycolysis and the P53 pathway, inflammation-related signatures like response to interferon alpha/gamma and TNF signaling, as well as angiogenesis. The two adjacent clusters of three models each showed comparably low enrichment scores in the mentioned hallmarks, but high scores in hallmarks G2M checkpoints, as well as E2F targets. The remaining PDX models showed individual expression profiles regarding the analyzed hallmarks, with three of the four PDX resembling the classical subtype grouping together.

Since we observed pronounced clustering in VEGF signaling and angiogenesis, the PI3K/Akt/mTOR pathway, and MAPK and JAK/STAT signaling (**Figure 4D**), all commonly deregulated in GBM, we analyzed expression of related gene sets in more detail. In VEGF and angiogenesis-related gene set analyses (**Figure S3A**), models resembling the mesenchymal subtype grouped together with two PDX mostly resembling the classical subtype, but with additional enrichment of the mesenchymal gene expression signature (Glio10535 and Glio13066). Another cluster of five models had comparably low enrichment of analyzed gene sets, while the remaining PDX showed different individual expression profiles. Clustering could also be observed in gene set enrichment analyses of PI3K/Akt/mTOR-related gene sets (**Figure S3B**), where four models were separated by comparably high enrichment scores, especially in PI3K/Akt dependent signaling. Another seven models showed comparable low enrichment scores for PI3K/Akt-dependent signaling, while the remaining 12 models showed different individual expression profiles regarding the analyzed gene sets. In analyses of MAPK/ERK and JAK/STAT signaling-related gene sets (**Figure S3C**), models of the mesenchymal subtype were again separate from the remaining models and characterized by overall high enrichment scores. The two PDX resembling the classical subtype the most pronounced (Glio10888 and Glio11368) were localized in a cluster of four models with comparably low enrichment scores, while remaining models showed individual expression profiles.

**Figure 5:**
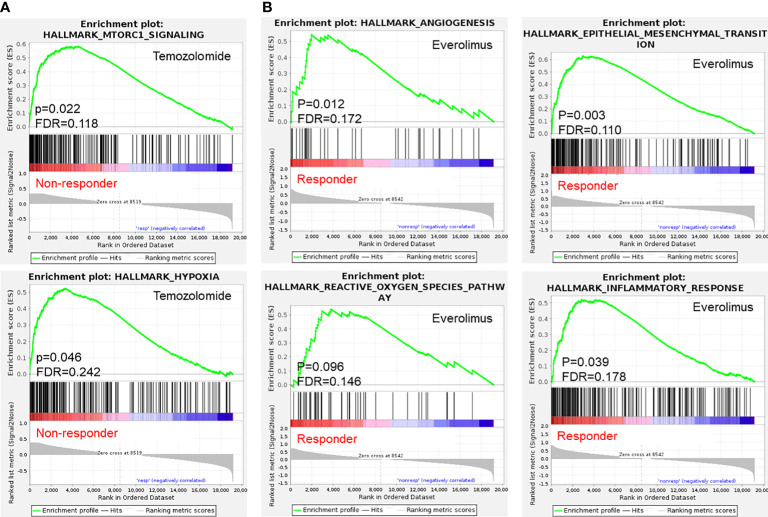
Selected gene set enrichment analysis plots of s.c. PDX tumor tissue based on results in chemosensitivity testing. **(A)** Enriched hallmark gene sets (p < 0.1 and FDR < 25%) in temozolomide resistant PDX and **(B)** PDX responding to mTOR inhibitor everolimus indicate possible implications of mTOR signaling and hypoxia for the monitored phenotypes.

In a next step we analyzed potential correlations between gene expression profiles and treatment responses of PDX tumors *via* gene set enrichment analyses. Comparison of expression profiles of irinotecan responders and non-responders did not reveal significant differences between the two phenotypes. Despite the observed clustering of PDX models in single-sample gene set enrichment analyses regarding their expressions of different VEGF signaling and angiogenesis-related gene sets, there was no statistically significant difference in between bevacizumab non-responders and responders. For temozolomide, our analyses did reveal significant differences (p < 0.1 and FDR < 25%) in expression profiles between responding and non-responding PDX (**Figure 5A**). In temozolomide resistant models, expression of hallmark gene sets hypoxia and mTORC1 signaling were upregulated, while no correlation between the expression of MGMT, a known predictive marker for temozolomide resistance (5, 6), and monitored responses could be detected. However, only one of these models were sensitive towards the mTOR inhibitor everolimus. Comparing expression profiles of everolimus responders and non-responders, responders showed higher scores for hallmark gene sets epithelial mesenchymal transition and angiogenesis, as well as in inflammatory response, TNF alpha signaling *via* NF-kappaB, IL6/JAK/STAT3 signaling and the reactive oxygen species pathway (**Figures 5B**, **S4**).

## 4 Discussion

There is an urgent need in preclinical research to identify and validate therapeutic alternatives for treatment of GBM and their subsequent clinical translation. Panels of PDX models that retain intertumoral heterogeneity and can approximate a cohort of cancer patients with individual chemosensitivities are therefore considered a valuable tool in drug testing and biomarker identification (20, 21, 38–40). In this study, we established and characterized 26 high-grade GBM PDX from mostly untreated primary patients, and established 15 matching orthotopic models. Histologically, PDX tumors showed comparable morphology and expression of MGMT and proliferation marker Ki-67 in comparison to the patients’ tumors and over a range of up to 11 *in vivo* passages. While s.c. PDX tumors exhibit nodular growth, orthotopic PDX models retain the typical infiltrative phenotype to varying degrees (19). Tumor doubling time of PDX models was heterogeneous but comparable over several subsequent s.c. passages, with their range reflecting intertumoral heterogeneity. At passage numbers > 10, we observed a drift towards increased tumor growth in individual models. This might be caused by stepwise adaption of the PDX tumor to the altered microenvironment in the immuno-compromised mice (41). Overall, our results confirm the stability of PDX models regarding their histology and growth characteristics, as seen in other panels of GBM PDX and of other tumor entities (19, 24, 39, 40, 42).

Extensive chemosensitivity testing of our PDX models revealed individual responses towards selected drugs. They included standard of care temozolomide, bevacizumab and irinotecan as drugs identified as beneficial in combination with temozolomide in subsets of patients, as well as compounds targeting different pathways identified as crucial in GBM biology, like mTOR inhibitor everolimus and multikinase inhibitor sorafenib. As expected, the standard of care temozolomide showed the best efficacy with 22 of 26 (85%) PDX models showing a response (T/C < 25%). This is higher than the up to 30% initial response seen in patients (43, 44), possibly in part due to the absence of the concentration limiting BBB (45). Irinotecan had an overall high, but compared to temozolomide, reduced efficacy with 3 out of 5 temozolomide-resistant PDX responding to irinotecan. Irinotecan as monotherapy and in combination with e.g. temozolomide and bevacizumab could not significantly improve outcome over temozolomide alone in various clinical trials (46–49), but still can be considered a treatment alternative for individual patients. However, the observed high initial sensitivity to temozolomide and irinotecan might be in part over-predictive due to different drug pharmacokinetics between humans and mice. Bevacizumab was clearly inferior regarding its anti-tumor efficacy in our panel. Tumor growth was inhibited in individual models, but no reduction of tumor size in any of our tested PDX was monitored. This is in line with various clinical trials showing that bevacizumab as first line monotherapy lacks sufficient efficacy but can improve progression-free survival in recurring GBM (50). As bevacizumab is specific for human VEGF, efficacy is also reduced in xenograft models where parts of the VEGF could be of murine origin. Our PDX panel furthermore confirmed the limited efficacy of the mTOR inhibitor everolimus currently used in clinical trials, despite the crucial role the PI3K/Akt/mTOR pathway in many GBM (13, 51). In 4 models however, we saw a moderate response. Sorafenib and salinomycin both showed anti-tumor activity in preclinical *in vitro* and *in vivo* studies (52–54), but not in clinical applications (55, 56). In our screenings we did not observe a tumor growth inhibition in any of the PDX. While this reflects the mentioned limited efficacy of these monotherapies, it remains unclear whether higher doses could achieve better results. Overall, our screening results reflect some of the response patterns seen in patients, and strongly indicate that intratumoral heterogeneity and clonal complexity is maintained, contributing to the monitored highly tumor individual sensitivity profiles. With subsequent gene set enrichment analyses, we tried to identify molecular marker profiles to identify patients that might benefit from personalized therapies.

As the tumor microenvironment has an important function in GBM development and proliferation, orthotopic models are the preferred setting for translational research. Comparing drug sensitivity between selected s.c. and matching orthotopic PDX models, we monitored a pronounced reduction of efficacy in the orthotopic setup. Irinotecan and temozolomide still caused inhibition of tumor growth, but to a lesser degree than in the respective s.c. PDX models. Both bevacizumab and everolimus performed notably worse, with no response in any of the tested orthotopic PDX. A possible reason for the reduced efficacy in orthotopic PDX is the BBB (57). It physically restricts diffusion of molecules into the brain and maintains a strict homeostasis *via* various efflux transporters. Both can drastically limit drug concentrations in the GBM (58), as observed in clinical studies with disappointing results in GBM patients, despite promising preclinical data (59, 60). Our results confirm that orthotopic PDX are a valuable tool to develop and test new therapy approaches that increase tissue selective drug delivery and efficacy while managing systemic side effects (61). However, as these orthotopic models are extremely time and cost sensitive, we suggest a two-step screening strategy for new therapies. In the first step, new compounds might be screened in s.c. transplanted models to identify potential active compounds. In a second step, one should test the selected compounds in orthotopic models to evaluate the potential effect of the BBB. Alternatively, one could transplant the GBM PDX in parallel s.c. and orthotopically to discriminate compounds affected by the BBB (62).

The available transcriptome sequencing data allowed us to analyze the PDX models mutation and gene expression status. We were able to confirm IDH-wt (R132) status in all models, with only one model bearing a mutation in IDH1, albeit not in codon R132. We furthermore found mutations frequently identified in patients’ GBM like in the genes EGFR, TP53, FAT1, as well as PTEN and MTOR (33).

Principal component analyses revealed three molecular subgroups within our IDH-wt GBM. They resemble previously described proposed molecular subtypes mesenchymal, classical and proneural regarding their expression profiles, reflecting the expression patterns and intratumoral heterogeneity seen in GBM patient cohorts (12). PDX models showed individual expression profiles of gene sets related to VEGF signaling and angiogenesis, and MAPK/Erk and STAT signaling, with comparably high expressions in the mesenchymal subtype. In PI3K/Akt/mTOR pathway-related gene sets, a majority of PDX showed enrichment in PI3K or mTOR signaling, or both. This well reflects the high prevalence of deregulation of this pathway seen in patients and its importance in GBM (63, 64).

Despite the observed clustering, for bevacizumab and irinotecan no relevant correlations between treatment response and gene expression profiles were detected. Gene set enrichment analyses of temozolomide response revealed high expression of hallmark gene sets for hypoxia and mTORC1 signaling in resistant models, while correlation between MGMT expression, a described predictive marker for response towards temozolomide, could not be identified (5, 6). It has been shown that a hypoxic microenvironment can induce the acquisition of temozolomide resistance *via* activation of HIF1-alpha signaling (65, 66), both mTOR dependent (67, 68) and independent (69, 70). Combination therapies of temozolomide and novel mTORC1/2 inhibitors for example have been successfully applied to overcome this temozolomide resistance *in vitro* (71–73). In a subgroup of patients in the EORTC trial 26082, activated mTOR signaling correlated with better treatment response towards monotherapy of mTOR inhibitor temsirolimus than to temozolomide (74). Our results strongly support these findings and could provide the basis for further analyses regarding the role of hypoxia and mTOR signaling in temozolomide resistance. Focus needs to be the evaluation of such combinations in orthotopic models since the altered microenvironment in the brain might impact the tumors response to hypoxia. In addtion, the BBB could limit the passage of different mTOR or PI3K inhibitors in the brain by varying degrees. Other *in vitro* studies however showed that mTOR inhibition under hypoxic conditions can promote survival of glioma cell lines *in vitro* by reducing oxygen and glucose consumption, and promotes temozolomide resistance by increasing MGMT protein levels (75, 76). These contrasting roles of mTOR signaling highlight the need for molecular markers to select GBM patients where hypoxia and mTOR signaling are true drivers of progression and temozolomide resistance. In our screening only 1 of 5 temozolomide resistant models was sensitive towards mTOR inhibition.

Enrichment of hypoxia-related hallmark gene sets, reactive oxygen species pathways and angiogenesis (67, 77) were also observed in everolimus responders and might be linked to the mesenchymal subtype, as indicated by enrichment of the hallmark gene set epithelial mesenchymal transition. Under hypoxic conditions, reactive oxygen species can accumulate and cause additional activation of HIF1-α, and thereby trigger various processes related to tumor progression, such as angiogenesis *via* expression of e.g., VEGFs and their receptors VEGFR1/2 (78–80). However, whether a combination therapy with e.g., bevacizumab would be beneficial in these GBM PDX needs further testing.

In conclusion, the enrichment of hypoxia and mTORC1 signaling in temozolomide resistant models, as well as enrichment of hypoxia-related gene sets in models susceptible for mTOR inhibitor everolimus indicate a role of mTOR signaling in progression under hypoxic conditions in s.c. GBM PDX (70). Whether combination with mTOR inhibitors can overcome temozolomide resistance in s.c. and matching orthotopic PDX needs further investigation. This includes analyzing expression profiles of orthotopic models to help understand biology and resistance formation in comparison to s.c. PDX. Gained insight might reveal further, yet unknown vulnerabilities for novel therapies.

Our results highlight both the possible applications, as well as limitations of the two model types. Subcutaneous PDX panels, while not fully representing the GBM’s tumor microenvironment, BBB, and their impact on treatment outcome, are suitable for larger screenings of new drugs or treatment regimens in a clinical study-like setup. Subcutaneous PDX panels can identify predictive markers, and mechanisms of intrinsic or treatment-induced drug resistance, and how to prevent or overcome it. Orthotopic GBM PDX - in a possible second step - enable analysis of the impact of the tumor microenvironment and the BBB on drug efficacy, their contribution to resistance formation, and whether drugs can reach therapeutically relevant concentrations within the tumor. This can be of particular interest in the development of new formulations or drug delivery systems that aim to increase BBB-crossing of available drugs in repurposing efforts and new drug candidates identified in previous screenings.

## Data availability statement

All underlying RNA sequencing data have been deposited at the European Genome-phenome Archive (EGA), which is hosted by the EBI and the CRG, under accession number EGAS00001007119. Further information about EGA can be found on https://ega-archive.org.

## Ethics statement

The animal studies were reviewed and approved by Landesamt für Gesundheit und Soziales, LaGeSo Berlin, Germany (H0308/18 and A0010/19).

## Author contributions

JA, JeH and WW designed this work. JoH, ML, TK, AJ, SK, CS and SK were responsible for patient tumor tissue collection and inclusion for the project. JA and AO supervised the experimental studies. JA, AO, MD and MB analyzed the data. JA, LW, WW and JeH wrote the manuscript. All authors contributed to the article and approved the submitted version.
