# Supplementary figures and images for: Clinically relevant glioblastoma patient-derived xenograft models to guide drug development and identify molecular signatures
Source: Front Oncol. 2023 Apr 11;13:1129627. doi: 10.3389/fonc.2023.1129627 (PMC10126369; doi:10.3389/fonc.2023.1129627)

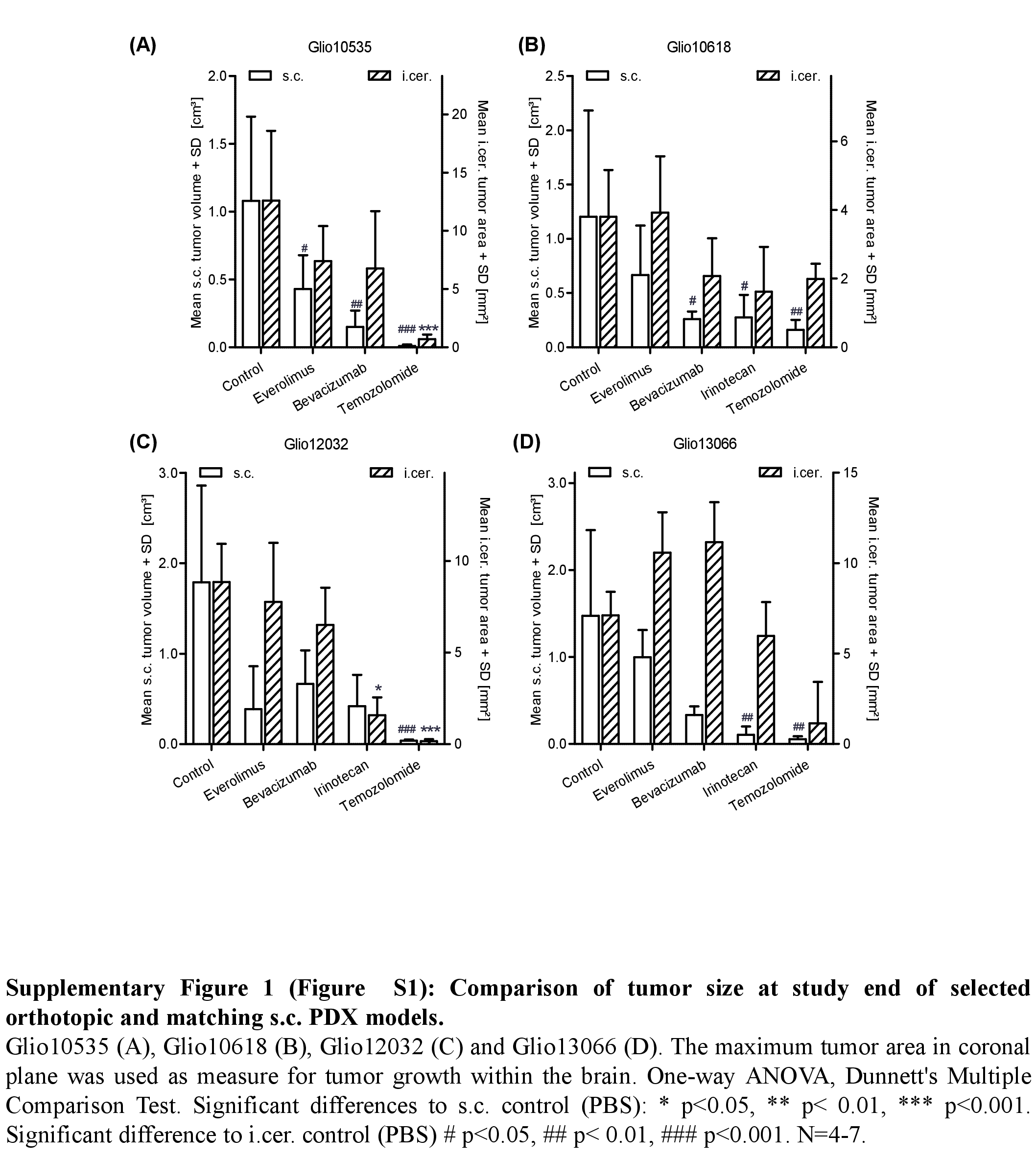

Supplement: Supplementary file 1 [file Image_1.tif]

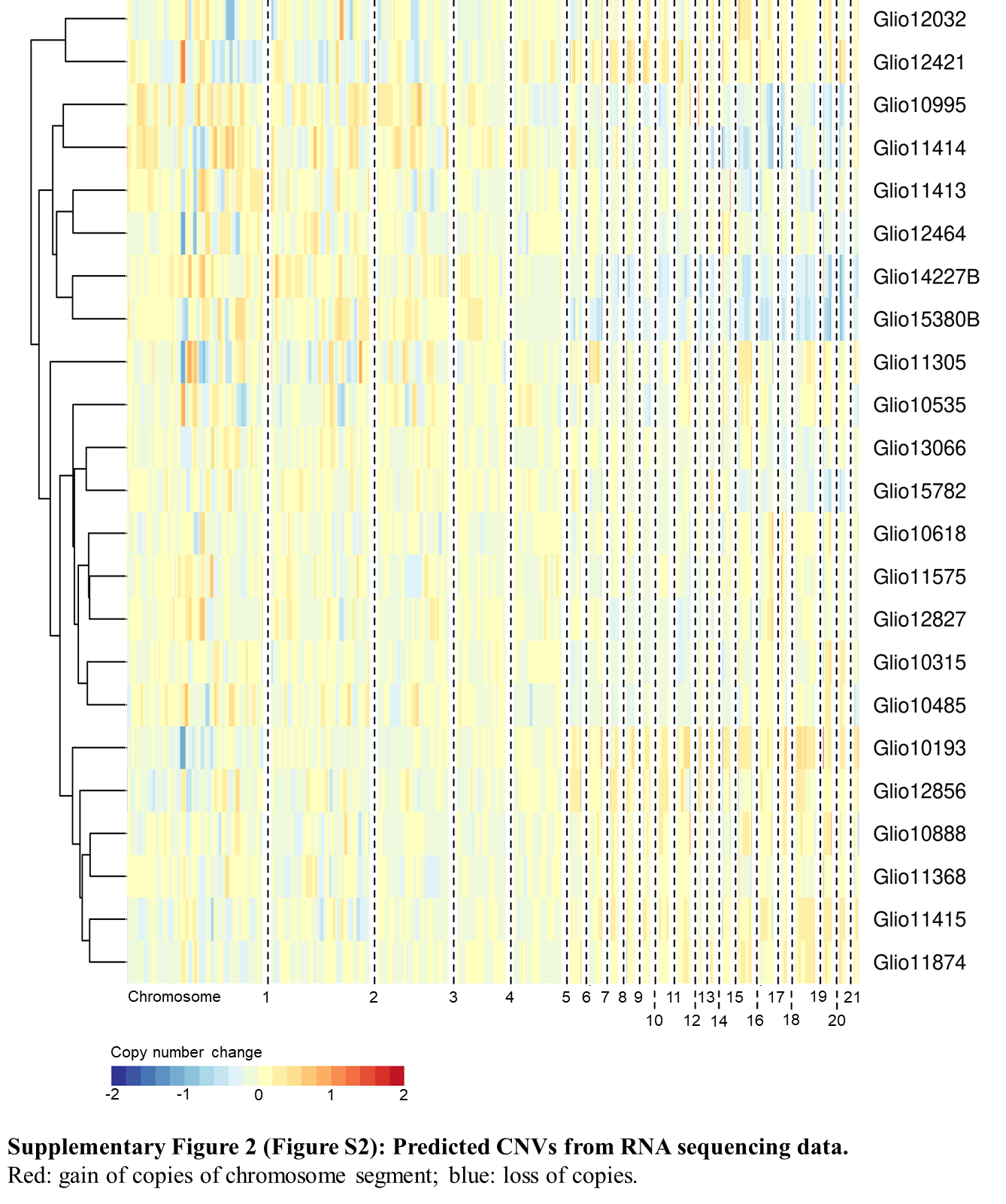

Supplement: Supplementary file 2 [file Image_2.tif]

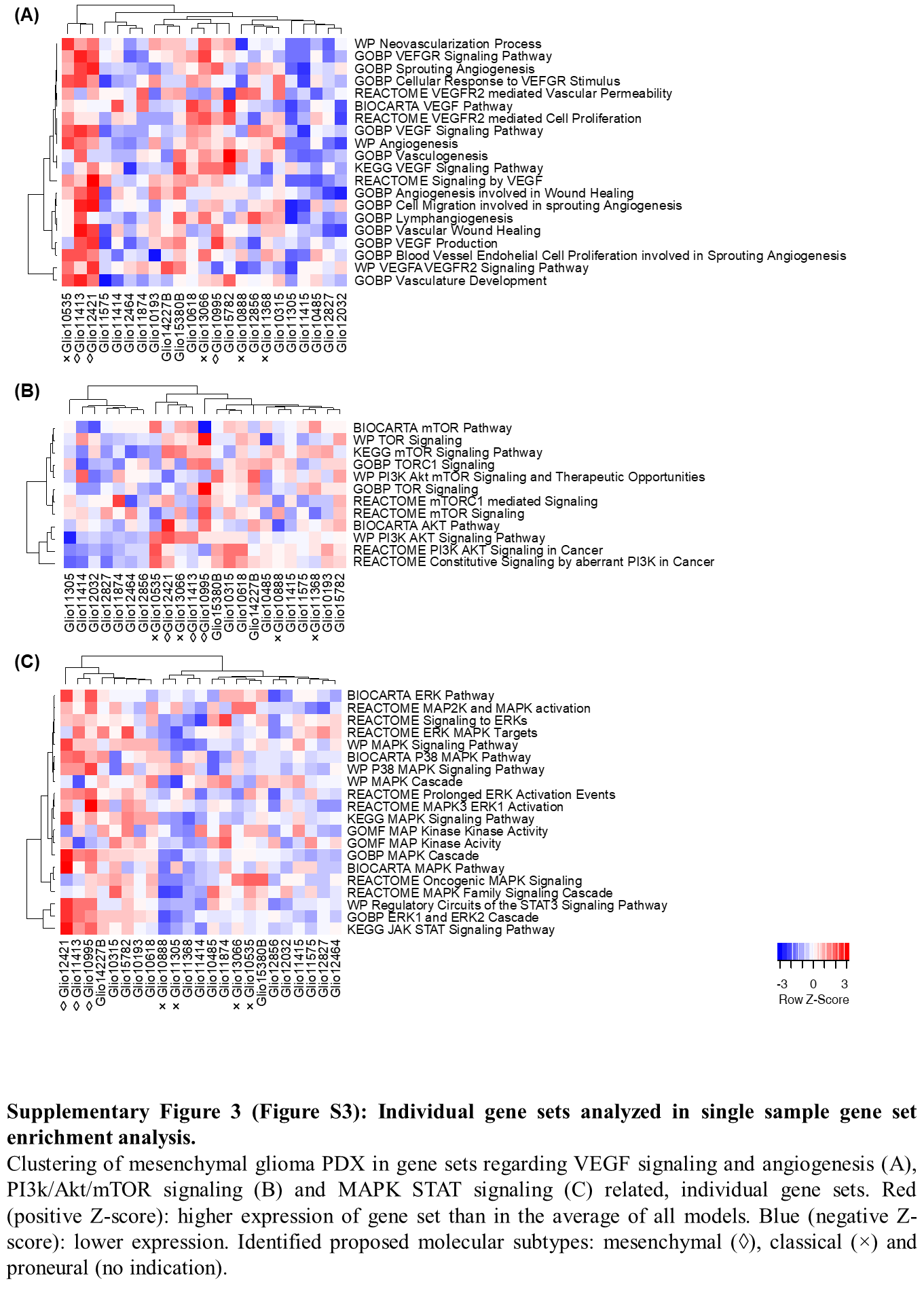

Supplement: Supplementary file 3 [file Image_3.tif]

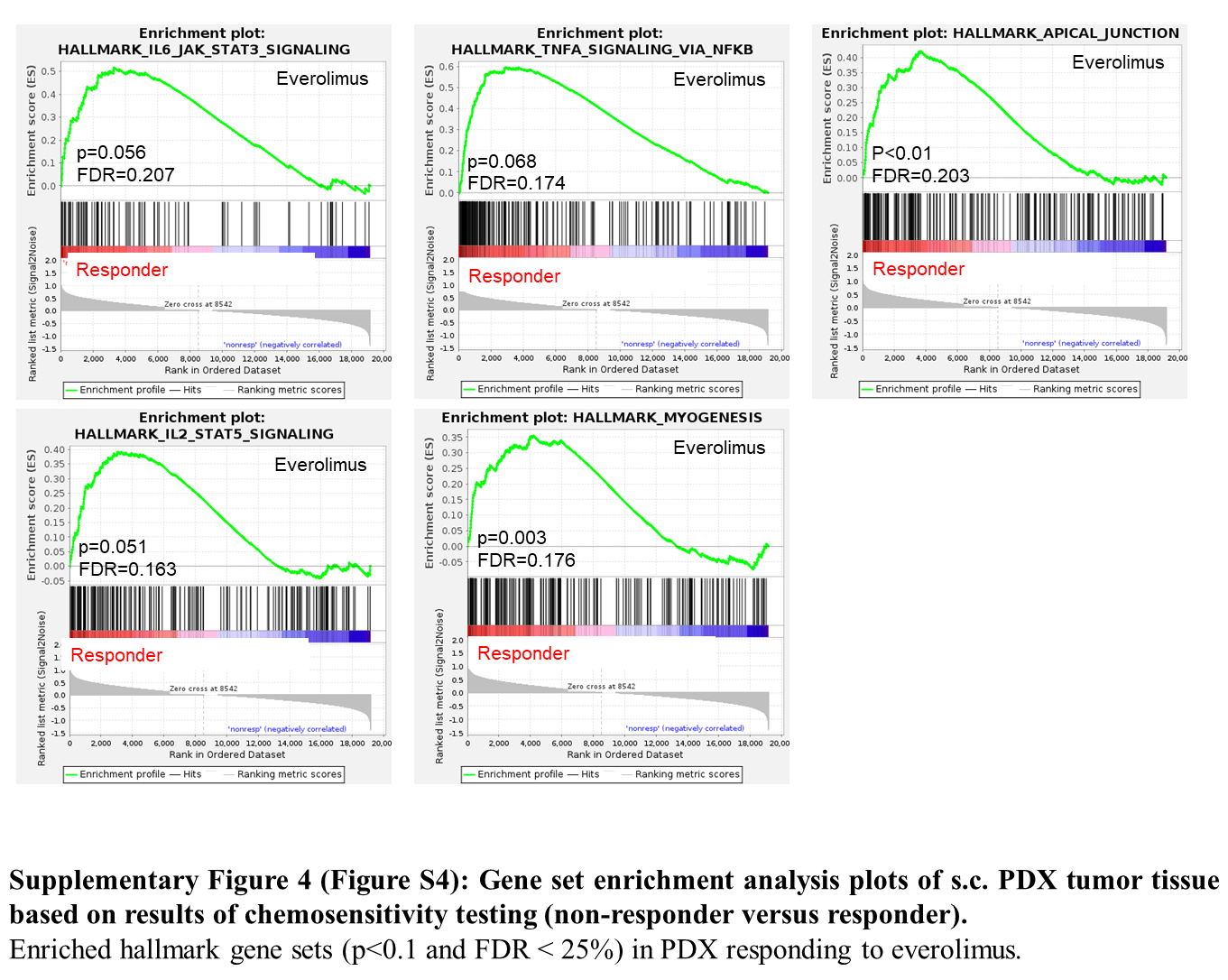

Supplement: Supplementary file 4 [file Image_4.tif]
